# PD-L1 is upregulated in CD163+ tonsillar macrophages from children undergoing EBV primary infection

**DOI:** 10.3389/fimmu.2022.940910

**Published:** 2022-11-14

**Authors:** Agustina Moyano, Natalia Ferressini, Elena De Matteo, Maria Victoria Preciado, Paola Chabay

**Affiliations:** ^1^ Molecular Biology Laboratory, Multidisciplinary Institute for Investigation in Pediatric Pathologies (IMIPP), Pathology Division, CONICET-GCBA, Ricardo Gutiérrez Children’s Hospital, Buenos Aires, Argentina; ^2^ Multidisciplinary Institute for Investigation in Pediatric Pathologies (IMIPP), Pathology Division, CONICET-GCBA, Ricardo Gutiérrez Children’s Hospital, Buenos Aires, Argentina

**Keywords:** EBV, macrophages, PD-L1, children, tumorigenesis

## Abstract

Epstein–Barr Virus (EBV) is a tumor associated virus that modulates not only the infected cells but also innate and adaptive immunity. Macrophages play a key role in tumor development and progression. Particularly, the M2 phenotype (CD163) with anti-inflammatory activity contributes to a favorable microenvironment for tumor development while the M1 (CD68) proinflammatory phenotype contributes to a restrictive one. In the context of pediatric EBV infection, little is known about macrophage contribution to PD-L1 expression, a molecule involved in immune exhaustion. We studied tonsils of primary infected (PI), healthy carriers (HC), reactivated (R), and not infected (NI) pediatric patients. Positive correlations were demonstrated for CD68+PD-L1+ in R and for CD163+PD-L1+ only in PI. Furthermore, CD163+PD-L1+ cell numbers were higher than PD-L1+CD68+ in PI patients. In addition, a positive correlation between PD-L1+CD163+ cells and LMP1 viral latent protein was observed in PI patients, and a positive correlation between PD-L1+CD68+ cells and BMRF1 lytic antigen was demonstrated. A positive correlation between TGF-β and PD-L1 expression was demonstrated in HC patients. Our findings indicate that EBV’s lytic and latent antigens might be regulating macrophages’ PD-L1 expression, particularly in PI patients, whereas, surprisingly, only TGF-β could be related to total PD-L1 upregulation. Given the relevance of macrophages and the PD-1/PD-L1 pathway in tumor progression and survival, more studies in early EBV infection could help to develop EBV-associated tumor therapies.

## Introduction

The Epstein–Barr virus (EBV) is a gamma-herpesvirus homogeneously distributed worldwide that infects more than 90% of the human population. EBV primary infection occurs in tonsils where the virus reaches the B lymphocyte, its target cell, and it persists life-long latent in the memory B cell as an episome under the control of the host’s immune system. As with all herpesviruses, after the primary infection, EBV establishes two different life-cycle programs, the latency and the lytic with a differential viral protein expression. During the lytic cycle, new infectious virions are produced allowing the virus to spread, while in latency, the virus persists silently in host cells avoiding immune recognition (1). In the majority of EBV-infected individuals, the virus coexists within the host without serious consequences. However, in a small number of cases, the development of EBV-associated neoplasia might occur (2). EBV was the first human virus to be related with oncogenic processes, since it has the ability to immortalize normal resting B lymphocytes *in vitro* and to establish lymphoblastoid cell lines. Moreover, EBV has been found in the neoplastic cells of numerous tumors, such as Burkitt’s lymphoma, NK/T-cell lymphoma, Hodgkin’s lymphoma (HL), non-HL, nasopharyngeal carcinoma, among others (3, 4). How a silent infection derives in malignancy and EBV’s contribution to this process are uncertain; in fact, viral gene expression widely differs between EBV-associated malignancies. However, each viral gene product may be determinant for viral oncogenicity. For instance, EBNA1 is responsible for viral episomal maintenance and it is not recognized by the immune system, which allows the virus to evade immune surveillance. What is more, LMP1 can activate intracellular signaling, modulating gene expression involved in proliferation, adhesion molecules, anti-apoptotic activities, and growth factors (5). In addition, lytic viral BCRF1 protein shows high homology to human IL-10, an anti-inflammatory cytokine that inhibits the activation and effector function of T cells, monocytes, and macrophages (6). In summary, EBV not only modulates the infected B cell but also the innate and adaptive immunity, creating a propitious environment for tumor development and progression. Our group previously reported the recruitment of macrophages surrounding EBV+ cells in tonsils (7). Even more, we described that, in EBV+ pediatric patients with a broader expression of latency antigens, the presence of alternative activated macrophages (M2) increased, although classically activated macrophages (M1) prevail in the entire cohort (8). Interestingly, when we studied pediatric EBV-associated HL, M1 macrophages prevailed in the tumor microenvironment as well (9). Besides these findings, little is known about the macrophages’ role in EBV infection and their contribution to lymphomagenesis. Macrophages belong to the innate immune system and have the ability to express distinct functional programs depending on micro-environmental signals; roughly, they can be polarized towards M1 (CD68+) or M2 (CD163+) profiles. The first ones are induced mostly by IFN-γ, have high antigen presentation capacity, have potent pro-inflammatory effectors, and are crucial in pathogens and tumor cell control and destruction. The second activation pathway (M2), mainly occurs under the stimulation of IL-4, IL-13, and IL-10 cytokines. These macrophages are involved in tissue repair, angiogenesis, and inflammation suppression through the secretion of cytokines such as TGF-β, IL-1R, and IL-8, creating an anti-inflammatory environment (10). The M2 phenotype shares several functionalities with macrophages found at tumor sites, known as tumor-associated macrophages (TAM). TAMs derive from peripheral blood monocytes attracted by the tumor *via* chemoattractants and cytokines that also act as mediators to regulate intracellular functions. TAMs are also responsible for inducing T regulatory cells rather than Th1. A microenvironment is crucial for tumor progression and invasion, as it has not only an active role in the recruitment of cells that empower the pro-tumoral conditions, but even more, it promotes the inhibition of those cells with antitumoral functions so as to escape from immunity (11). One well-described evasion mechanism is immune exhaustion through programmed cell death protein 1 (PD-1) and its ligand PD-L1, an immune checkpoint that induces immune tolerance on T cells. In fact, the activation of this axis can negatively impact T-cell-mediated immunity, induce T cell apoptosis, lead to functional failure and IL-10 production, and promote Tregs differentiation, protecting tumor cells from cytotoxicity (12). Upregulation of PD-1 and PD-L1 was described in a large number of tumors. In a previous study, our group demonstrated an upregulation of PD-L1 expression exclusively at the microenvironment of pediatric EBV-associated Hodgkin lymphomas (HL) (13). Furthermore, in adult HL, it was described that PD-L1 in the tumor microenvironment (TME) is mostly expressed by TAMs (14), but it was not explored in EBV-associated cases.

In Argentina, the analysis of different pediatric lymphomas associated with EBV (Hodgkin, Burkitt and Diffuse Large B cell lymphoma) revealed a higher prevalence in children younger than 10 years old (15). Given that the immune response to EBV infection is significantly different between adults and children, considering that in Argentina the incidence of EBV-associated tumors increases when it comes to pediatric patients, and that the role of macrophages in the control of EBV infection and tumorigenesis is still poorly explored, our aim was to evaluate the influence of EBV in macrophages’ PD-L1 expression in pediatric patients to more deeply understand its viral contribution to lymphomagenesis.

## Materials and methods

### Ethical statement

All samples were collected after written consent (for patients older than 12 years and legal guardians of children younger than 12 years) and assent (7- to 12-year-old patients and legal guardians of children older than 12 years) were obtained, following the national and international ethics standards and under the supervision of the Ethical Committee of the Ricardo Gutiérrez Children’s Hospital, in accordance with the Helsinki Declaration of 1975.

### Patients and samples

Fresh tissue samples from patients undergoing tonsillectomy due to nonreactive hyperplasia, diagnosed according to international routine protocols for recurrent chronic inflammation at the Otorhinolaryngology Division, Ricardo Gutierrez Children’s Hospital (Buenos Aires, Argentina), were collected. For the surgery to be performed, patients must be completely asymptomatic, without signs or symptoms of acute inflammation or infection such as fever, sore throat, or cough. At surgery, a concomitant blood sample was obtained and serum was separated to determine the EBV infection status using indirect immunofluorescence assay, as previously reported (16). A portion of the tissue was preserved at −70°C for later nucleic acid extraction, and the remaining biopsy tissue was formalin-fixed paraffin-embedded (FFPE) at the Pathology Division of Ricardo Gutierrez Children’s Hospital (Buenos Aires, Argentina). For this study, a cohort of 36 patients aged between 1 and 15 years (median age of 5 years), were analyzed. The cohort included 11 primary infected (PI), 10 healthy carriers (HC), 11 undergoing reactivation (R), and four not infected (NI) patients. These patients’ infectious status were determined according to the presence or absence of different EBV sera antibodies (VCA-IgM, VCA-IgG, early antigen (EA)-IgG and EBNA1-IgG), as reported (17). Primary infected patients (PI) were defined as VCA-IgM+/VCA-IgG-/+/EA-IgG-/EBNA1-IgG-; healthy carriers (HC) as VCA-IgM-/VCA-IgG+/EA-IgG-/EBNA1-IgG+; patients undergoing viral reactivation (R) were defined as VCA-IgM+/-/VCA-IgG+/EA-IgG+/EBNA1-IgG+; and non-infected patients (NI) as VCA-IgM-/VCA-IgG-/EA-IgG-/EBNA1-IgG- (**Supplementary Table 1**).

### Immunohistochemistry/immunofluorescence

Immunohistochemistry (IHC) was performed on serial cuts of the FFPE biopsies (3–4 µm). For viral antigens, LMP1 (CS1-4 pool of clones, Dako) and EBNA2 (1E6 y R3 clones, Abcam) primary antibodies as latency antigens and the BMRF1 primary antibody (G3-E31 clone, Abcam) as an early lytic antigen were used as described (16). Cytokine detection was assessed with specific primary antibodies against IFN-γ (polyclonal, Abcam), IL-10 (Ab34843, Abcam), and TGF-β (Ab9758, Abcam) as described (8). Lastly, a double-staining for macrophage polarization markers CD68 (clone KP-1, Roche Ventana), CD163 (clone MRQ-26, Roche Ventana), and PD-L1 (clone 4E54, Abcam) was accomplished. Antigenic retrieval was performed using sodium citrate buffer pH = 6.0. After incubation with the first primary antibody (CD68 or CD163), immunodetection was performed using a commercial kit (ScyTek) followed by diaminobenzidine (DAB) as a chromogen. Then, the second primary antibody PD-L1 diluted in 1/100 TBS-BSA 2% was incubated overnight, and antibody signal amplification was performed using Alexa 594 anti-mouse, followed by Hoescht.

### Real time-PCR

To complete the cytokine expression panel, IL-1β and TNF-α were evaluated previously in this series by RT-PCR as described (8).

### Image acquisition and quantification

Tonsils were divided principally in two histological regions in which well-described processes occur. To evaluate the differences between regions and to best represent the overall tissue, for each patient, 10 pictures of the interfollicular region (IF) and 10 of the germinal center (GC) were taken at 400X and 1000X, using the ZEN 2 (blue edition) imaging platform, with A1 AxioScope, Carl Zeiss microscope. For IL-10, IFN-γ, and TGF-β, positive cell count was performed, and results were expressed as positive cells per area unit (cells+/mm^2^). For the double staining, ImageJ imaging program was used. First, the cell counts of individual markers were made (CD68, CD163, PD-L1), then pictures were merged and double-positive cells were counted (CD68+PD-L1+ and CD163+PD-L1+). Afterwards, positive cells were averaged and expressed per area unit (cells+/mm^2^). For viral proteins, cell count was assessed in the whole tissue section and expressed as positive cells per area unit (cells+/mm^2^) as well.

### Statistical analysis

Data were analyzed using the GraphPad Prism 8 software. Normality test was performed using Shapiro-Wilks’s test. Comparison between groups was assessed by one-way ANOVA or Kruskal-Wallis tests according to normality test results. Correlations were performed using Spearman or Pearson tests. Categorical variables were analyzed with the Fisher exact test or chi-square test. Outliers were defined using the Robust test to compare data median absolute deviation (Mad) on Excel. All tests were two-tailed, and p < 0.05 was considered statistically significant.

## Results

### PD-L1 distribution

**Figure 1:**
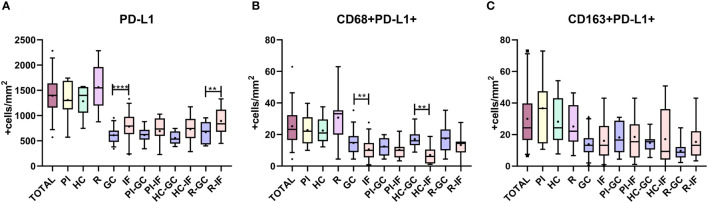
Histological distribution of PD-L1. **(A)** Total PD-L1 expression in the germinal center (GC) and interfollicular region (IF) in the entire cohort (Total), in primary infected patients (PI), healthy carriers (HC), and patients undergoing reactivation (R); **(B)** CD68 macrophages’ PD-L1 expression in the germinal center (GC) and interfollicular region (IF) in the entire cohort (Total), in primary infected patients (PI), healthy carriers (HC), and patients undergoing reactivation (R); **(C)** CD163 macrophages’ PD-L1 expression in germinal center (GC) and interfollicular region (IF) in the entire cohort (Total), in primary infected patients (PI), healthy carriers (HC), and patients undergoing reactivation (R) **p < 0.01, ****p < 0.0001.

Our group previously described a recruitment of PD-L1+ cells at the microenvironment of EBV-associated pediatric lymphomas (13). Therefore, PD-L1 expression was evaluated in tonsils in a cohort of pediatric patients infected with EBV. Neither significant differences were observed in the PD-L1+ cell count between infection status (PI, HC, and R), nor between EBV+ and EBV- patients (p > 0.05, *t*-test). Regarding histological distribution, between interfollicular (IF) and germinal center (GC) regions, PD-L1 expression was significantly higher in the IF region when we analyzed the whole series (p < 0.0001, *t*-test), and it was particularly sustained in R (p = 0.0021, *t*-test) (**Figure 1A**).

### PD-L1 and macrophages

**Figure 2:**
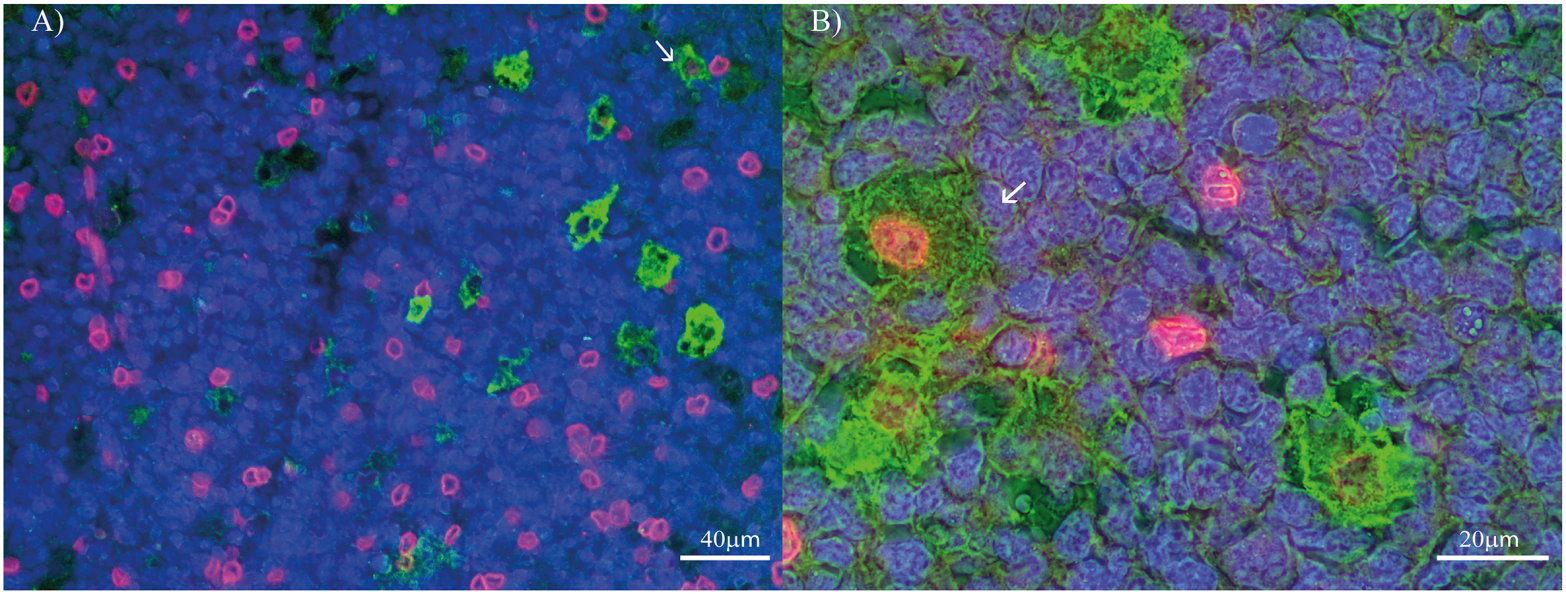
PD-L1 and macrophages’ polarization profile markers double-staining in pediatric patients’ tonsil. **(A)** Double stain for PD-L1 (Red) and CD68 (Green) at 400X. **(B)** Double stain for PD-L1 (Red) and CD163 (Green) at 1000X. Each image includes a nuclear counterstain Hoechst (blue).

**Figure 3:**
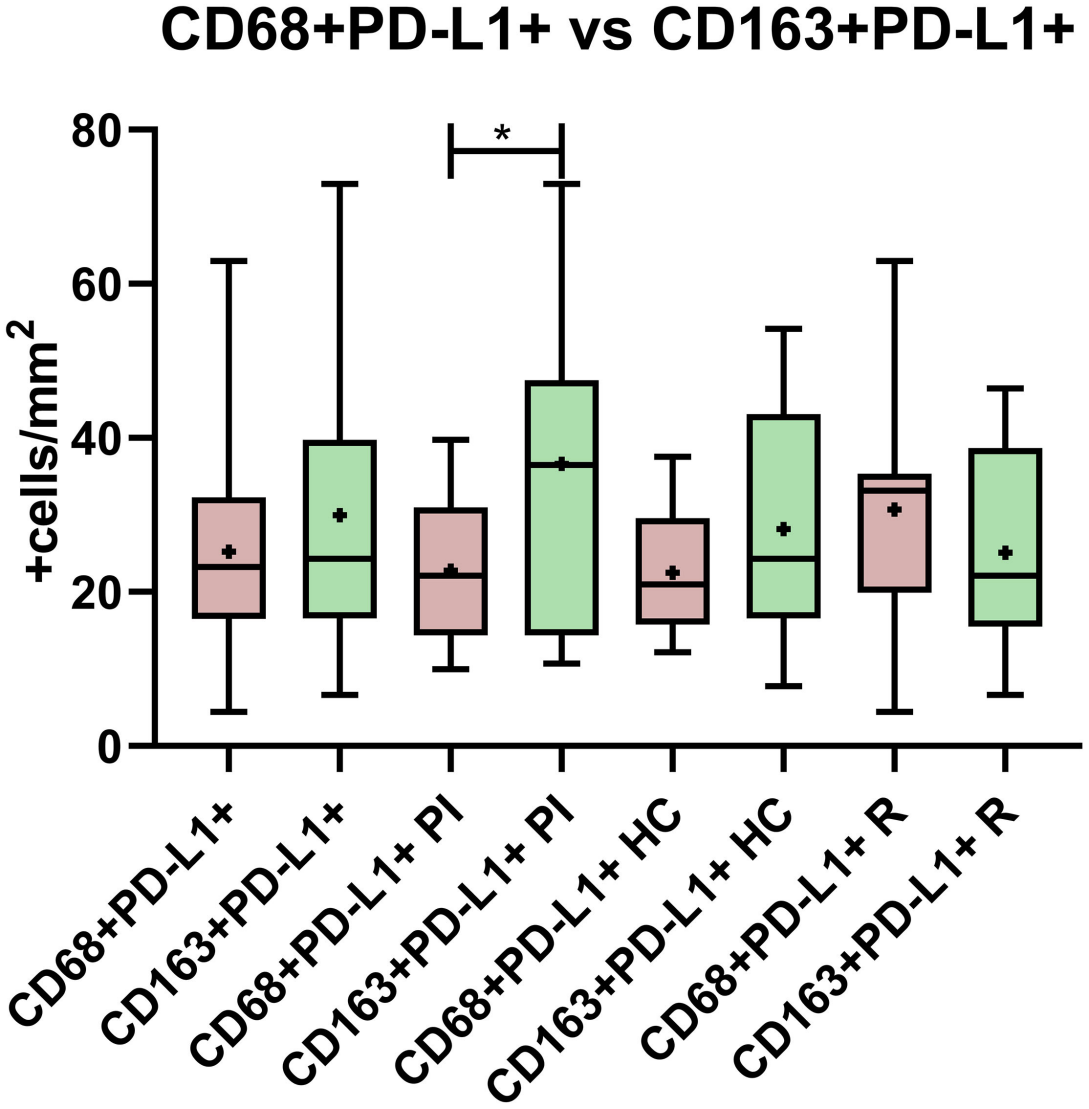
PD-L1 expression comparison between CD68 and CD163 macrophages in the entire cohort, in primary infected patients (PI), healthy carriers (HC), and patients undergoing reactivation (R). *p < 0.05.

Since it was described that PD-L1+ cells around Hodgkin Reed Sternberg (HRS) cells were macrophages in adult HL (14), the relation between PD-L1 expression and macrophages’ phenotype was explored. Double-positive staining, namely, CD68+PD-L1+ or CD163+PD-L1+, was evaluated for each viral infectious status, and no significant differences among PI, HC, and R as well as between EBV+ vs. EBV- were demonstrated (p > 0.05, ANOVA and *t*-test) (**Figures 1B, C**, **2**). However, CD68+PD-L1+ cell count in R and CD163+PD-L1+ in PI seemed to be higher compared to the remaining infectious status. Therefore, correlations between total PD-L1+ cells and CD68+PD-L1+ or CD163+PD-L1+ cells were evaluated. Positive correlations were demonstrated for CD68+PD-L1+ only in R (r = 0.7546; p = 0.0073, Pearson) and for CD163+PD-L1+ only in PI (r = 0.6775; p = 0.022, Pearson). Furthermore, CD68+PD-L1+ was statistically higher in GC in the entire cohort (p = 0.0024, *t*-test), and in particular in HC (p = 0.0095, *t*-test), whereas no differences were observed when CD163+PD-L1+ cell counts were compared between GC and IF histological regions (p > 0.05, *t*-test). Finally, in order to explore whether PD-L1 expression prevailed in M1(CD68+) or M2 (CD163+) macrophages, we compared double-positive stain cell count between macrophages’ phenotypes, resulting in CD163+PD-L1+ cell numbers being higher than PD-L1+CD68+ specifically in PI patients (p = 0.0356, *t*-test) (**Figure 3**), while no significant differences were demonstrated in HC or R. Interestingly, when this analysis was performed considering both histological regions, in the IF region CD163+PD-L1+ cells was higher than PD-L1+CD68+ cells in the entire cohort (p = 0.0051, Wilcoxon), and CD68+PD-L1+ was higher in GC specifically in R (p = 0.0052, *t*-test).

### PD-L1 and cytokine environment

Macrophages have a great capacity of plasticity and the environment surrounding them is usually decisive for the direction in which they polarize. In a previous work, we studied the macrophages polarization environment in EBV infection, describing some interesting findings (8). Here, the interplay of cytokines IFN-γ, IL-10, TGF-β, IL-1β, and TNF-α with total PD-L1 and PD-L1 expressed by macrophages was evaluated. We found that the total PD-L1+ cell count negatively correlated with IFN-γ only in the R subgroup (r = -0.6404; p = 0.0387, Spearman) and positively with TGF-β only in HC (r = 0.6444; p = 0.0494, Spearman), and no significant correlations were observed for IL-10, TNF-α, or IL-1β (p > 0.05, Spearman). Unexpectedly, no correlations could be demonstrated for CD68+PD-L1+ or CD163+PD-L1+ with the explored cytokines (p > 0.05, Spearman).

### PD-L1 and viral protein expression

**Figure 4:**
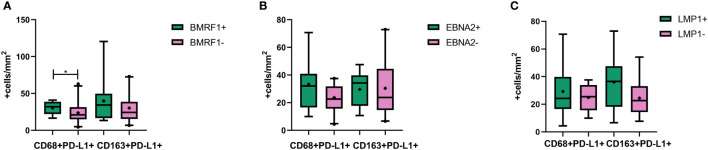
CD68 and CD163 macrophages’ PD-L1 expression in **(A)** the presence and absence of BMRF1 lytic viral protein, **(B)** the presence and absence of EBNA2 latent viral protein, and **(C)** the presence and absence of LMP1 latent viral protein. *p < 0.05.

Considering the different antigens expressed in each stage of the viral cycle, lytic and latent patterns were defined by IHC as follows: Latency 0, EBERs-/LMP1-/EBNA2-; Latency I, EBERs+/LMP1-/EBNA2-; Latency II, EBERs+/LMP1+/EBNA2-; and Latency III, EBERs+/LMP1+/EBNA2+ (**Supplementary Table 2**). To identify cells undergoing the lytic cycle, we evaluated the expression of the early nuclear antigen BMRF1 also by IHC. Viral protein expression was evaluated to investigate the role of the EBV latent and lytic antigens in macrophages’ contribution to PD-L1 expression in the context of different infection statuses. First, we evaluated the global PD-L1 expression, and no correlation with latent LMP1+, EBNA2+, or lytic BMRF1+ cell counts was found (p > 0.05, Spearman). Then, to further explore PD-L1 macrophages’ expression, correlation analyses were performed with CD68+PD-L1+ and with CD163+PD-L1+ cells. These analyses revealed that, in patients expressing BMRF1 lytic antigen, the CD68+PD-L1+ cell count was higher than in those cases with no BMRF1 expression (p = 0.0048, Mann–Whitney) (**Figure 4**). To add more, the BMRF1 cell count positively correlated with CD68+PD-L1+ cell counts (r = 0.3949; p = 0.03, Spearman). Finally, only in R, EBNA2+ cells positively correlated with the CD68+PD-L1+ cell count (r = 0.6226; p = 0.0448, Spearman) and, remarkably, only in PI, LMP1 positively correlated with CD163+PD-L1+ cells (r = 0.7517; p = 0.0267, Spearman).

## Discussion

Currently, the PD-1-PD-L1 immune-checkpoint (IC) is taking a great relevance as a target for immunotherapy in a large number of cancers, since blocking this pathway leads to antitumor responses with the consequent clinical benefit for some patients. The expression of PD-1 is upregulated on activated T-cells to regulate the immune response; when PD-1 engages its ligand PD-L1, the inhibitory signals occur, and immune tolerance is induced. Different tumor cells proved to overexpress PD-L1, generating an evasion mechanism; in fact, even microenvironment cells such as macrophages and dendritic cells, among others, have been described to upregulate PD-L1 expression as well. Therefore, great efforts have been made to deeply study and comprehend the pathway (18).

On the other hand, EBV has been widely studied for its oncogenic skills, and it has been associated with numerous lymphoid and epithelial tumors. Furthermore, the upregulation of PD-L1 in EBV-associated tumors has been described (19). Yet, the process by which a malignancy develops and the EBV contribution to lymphomagenesis are still not completely understood.

In Argentina, the incidence of lymphomas associated with EBV is higher in children younger than 10 years old (15), thus evaluating the behavior of the virus in healthy children, a population with increased susceptibility of developing viral-associated lymphomas, was the strategy to elucidate the first steps of the lymphomagenesis process and the EBV contribution to it. The increase of PD-L1 expression in natural killer/T-cell lymphoma tumor cells was related to the viral LMP1 antigen, since PD-L1 expression positively correlates with its expression (20). In addition, a possible correlation between the LMP2A latent viral protein and PD-L1 induction was suggested in Burkitt lymphoma with a non-canonical latency program (21). Also, the PD-L1 targeting oncosuppressor microRNA miR-34a was downregulated in EBNA2-transfected lymphoma cells *in vitro* (18). Moreover, in tonsils from adults with infectious mononucleosis (IM), PD-L1 expression was observed in EBV+ cells, although the bulk of PD-L1+ cells were EBV- (22). Those findings suggest that EBV could be involved in the regulation of this ligand expression. In pediatric HL from Argentina, Jiménez et al. demonstrated that, even though genetic alteration did not play a key role in this upregulation, the PD-L1+ cell count was higher at the microenvironment of EBV-associated cases (13). Moreover, the upregulation of PD-L1 expression by macrophages surrounding HRS tumor cells was demonstrated (14). In addition, it was demonstrated that EBV-transformed B cells exhibit strong immunoregulatory properties (23) which may contribute to inducing PD-L1 expression. Based on those findings, and since our group reported viral protein presence in tonsillar macrophages (24) as well as an immunoregulatory environment in EBV infection, particularly in PI, our aim was to disclose if EBV could trigger PD-L1 expression in macrophages in the context of primary infection or during viral persistence. As far as we know, this is the first work that characterizes macrophages’ contribution to PD-L1 expression in the context of EBV infection at the tonsils, the site of viral entrance and replication, of pediatric patients.

Unexpectedly, in our cohort, no significant differences in PD-L1+ cell numbers were observed between EBV+ and EBV- patients, and even among infection statuses (PI, HC, and R), the expression was homogeneous. What’s more, when we analyzed viral protein expression, no correlation with PD-L1 cell count was demonstrated. This preliminary finding may indicate that neither EBV primary nor persistent infection induces PD-L1 upregulation, quite the opposite to what was demonstrated in EBV-associated tumors.

To further explore PD-L1 expression in our cohort, we compared the double-staining cell count among infectious statuses and no significant differences were observed. However, when comparing the means between the CD68+PD-L1+ and CD163+PD-L1+ cell counts specifically in PI, HC, and R, the expression of CD68+PD-L1+ in the R group and CD163+PD-L1+ in PI seemed to be higher, though no statistical significance could be demonstrated. Remarkably, positive correlations with PD-L1 were demonstrated in both cases suggesting that CD163+ macrophages, as M2 markers, and CD68+ macrophages, as M1 markers, could play a role in PD-L1 expression in PI and R patients, respectively. Delving into these observations, we compared the CD68+PD-L1+ vs. CD163+PD-L1+ cell counts, and we evidenced that only in PI CD163+PD-L1+ was higher than CD68+PD-L1+. PD-L1 upregulation in M2 macrophages was previously suggested in EBV+ and EBV- HL (25), as well as in EBV-associated Burkitt lymphoma (21). Therefore, based on our observations, the upregulation of PD-L1 expression in M2 macrophages might be an early event in the context of primary EBV infection.

Macrophages have great plasticity; their polarization toward a proinflammatory (M1, CD68+) or an anti-inflammatory (M2, CD163+) phenotype depends on the interaction with the microenvironment. In a context of IFN-γ stimuli, polarization typically results in the M1 phenotype; in contrast, an IL-4, IL-10, IL-13, or TGF-β environment usually polarizes macrophages toward the M2 phenotype which is mainly involved in tissue repair, angiogenesis, and inflammation suppression through the secretion of cytokines such as TGF-β, IL-1R, and IL-8 (26). We analyzed correlations of double-stained positive cells with cytokines in the tonsil’s microenvironment in the context of EBV infection to evaluate if they were involved in macrophages’ PD-L1 expression. Unexpectedly, no correlations were demonstrated for CD68+PD-L1+ or CD163+PD-L1+ and cytokines. Therefore, the cytokines involved in polarization may not be as determinant as expected in PD-L1 upregulation in macrophages. Surprisingly, when total PD-L1 expression was evaluated, no correlation was observed between PD-L1 and IFN-γ, even though PD-L1 upregulation by this cytokine was reported in EBV-associated tumors (27, 28). Furthermore, when we evaluated correlations of cytokines in the different infectious status, a negative correlation between PD-L1 with IFN-γ was proven in the R subgroup. In contrast, TGF-β expression positively correlated with PD-L1 only in HC, suggesting that this regulatory cytokine could have a role in PD-L1 upregulation in the context of EBV persistent infection in tonsils, given that we previously described latency II and III viral antigens in this group of patients (8).

A peculiar behavior of macrophage phenotype expressing PD-L1 was observed in PI and R patients, and specific cytokine correlations with PD-L1 were seen in R and HC. In addition, we previously demonstrated an increase in CD163 cells in the context of viral latency II and III antigen expression and its correlation with EBNA2 cells (8). Therefore, we hypothesized that the viral antigens could be involved in PD-L1 upregulation in polarized macrophages. To achieve this purpose, correlations between viral proteins and CD68+PD-L1+ or CD163+PD-L1+ cell count were evaluated. In line with the previous results, exclusively in PI, the LMP1 antigen positively correlated with CD163+PD-L1+ cells. Therefore, we hypothesized that LMP1 may be involved in PD-L1 upregulation in M2 macrophages in PI. Besides, we found that CD68+PD-L1+ cell counts were higher in BMRF1+ cases. To add more, they also positively correlated with the BMRF1 lytic antigen in the entire cohort and with EBNA2 viral protein in R status, prompting us to suggest that in these patients PD-L1 expression could be upregulated to counteract and regulate the effect of pro-inflammatory M1 macrophages, contributing to a more permissive milieu for viral antigen expression. In light of these findings, we believe that more studies about the interaction of these and other viral proteins with macrophages are needed to better understand if viral latent and/or lytic proteins are involved in the regulation of PD-L1 expression. The studies in early events of EBV infection could help to develop more and better knowledge about the mechanism of viral association with tumor development.

## Data availability statement

The original contributions presented in the study are included in the article/**Supplementary Material**. Further inquiries can be directed to the corresponding author.

## Ethics statement

The studies involving human participants were reviewed and approved by Comite etica en investigación (CEI), Hospital de Niños Ricardo Gutierrez. Written informed consent to participate in this study was provided by the participants’ legal guardian/next of kin.

## Author contributions

Conceptualization, PC and AM; Methodology, AM; Formal Analysis, AM; Investigation, AM and NF; Resources, PC and EM; Data Curation, AM.; Writing-Original Draft Preparation, AM and PC; Writing-Review & Editing, PC and MVP; Supervision, PC; Project Administration, PC; Funding Acquisition, PC. All authors contributed to the article and approved the submitted version.

## Funding

This study was supported in part by a Grant from the National Agency for Science and Technology Promotion (PICT 2017 no. 1554, PICT 2015 no. 1533 and PICT 2018 no. 0966).

## Acknowledgments

We thank our patients and their families who agreed to participate in this study: Barbara Cao, Silvana Romero, Cristina Pabes, and Maria Jose Andrade (HistopathologicalLaboratory, at the Ricardo Gutierrez Children’s Hospital) for their helpful histotechnician work; and the Otorhinolaryngology Service at the Ricardo Gutierrez Children’s Hospital, which kindly helped us and provided us with the samples. PC and VP are members of the National Research Council (CONICET), Research Career Program, and MA and NF are CONICET doctoral fellows.

## Conflict of interest

The authors declare that the research was conducted in the absence of any commercial or financial relationships that could be construed as a potential conflict of interest.

## Publisher’s note

All claims expressed in this article are solely those of the authors and do not necessarily represent those of their affiliated organizations, or those of the publisher, the editors and the reviewers. Any product that may be evaluated in this article, or claim that may be made by its manufacturer, is not guaranteed or endorsed by the publisher.

## Supplementary material

Supplementary Table 1 Infection status by EBV serological profile.

Supplementary Table 2 Lytic and latency pattern expression in infection status. On the upper row is shown the number of patients expressing lytic viral protein regardless of the latency pattern.
