# Supplementary figures and images for: PD-L1 is upregulated in CD163+ tonsillar macrophages from children undergoing EBV primary infection
Source: Front Immunol. 2022 Nov 14;13:940910. doi: 10.3389/fimmu.2022.940910 (PMC9701750; doi:10.3389/fimmu.2022.940910)

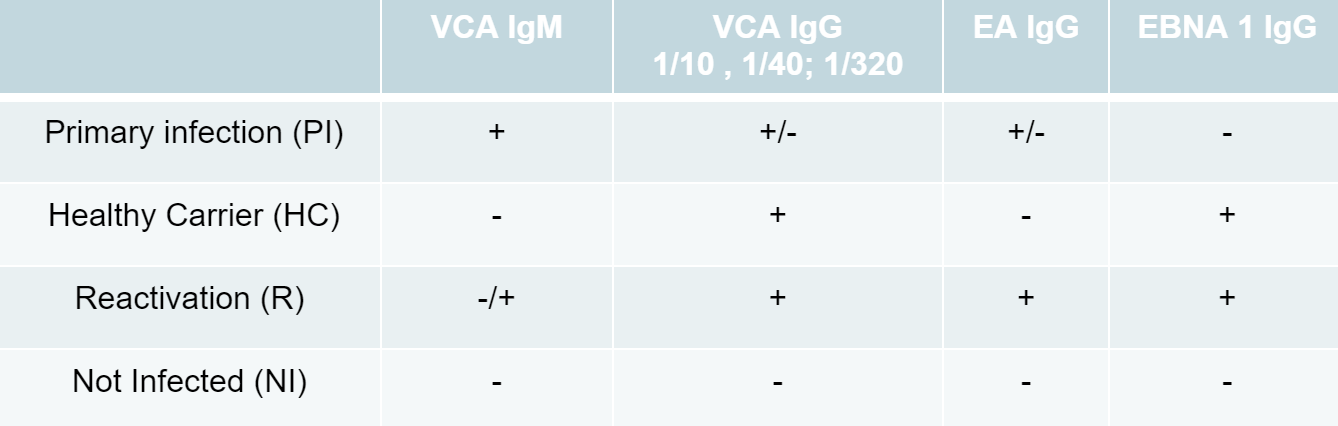

Supplement: Supplementary Table 1 — Infection status by EBV serological profile. [file Image_1.tiff]

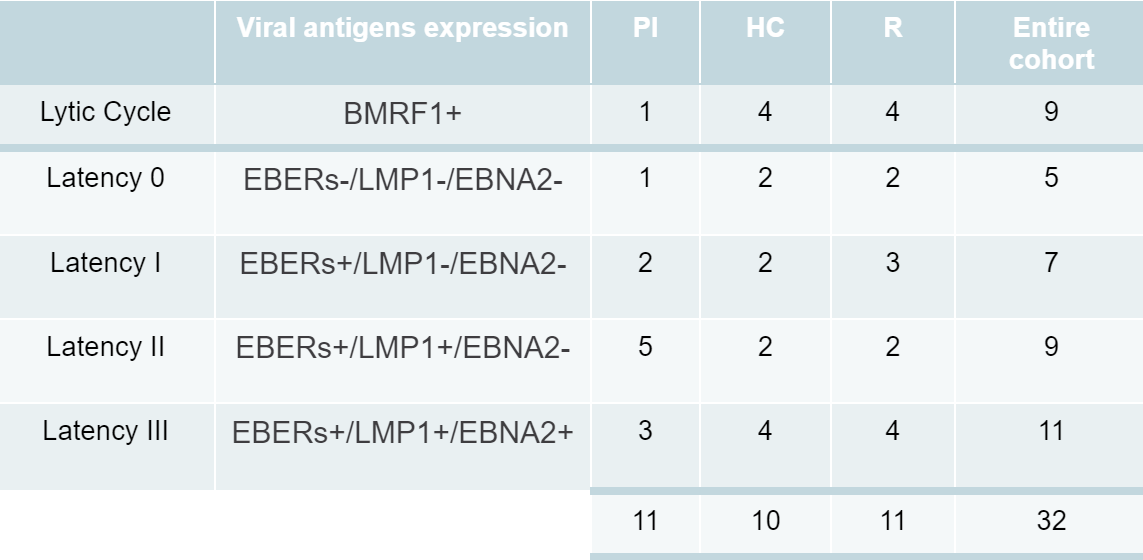

Supplement: Supplementary Table 2 — Lytic and latency pattern expression in infection status. On the upper row is shown the number of patients expressing lytic viral protein regardless of the latency pattern. [file Image_2.tiff]
